# Supplementary material for: A prebiotic template-directed peptide synthesis based on amyloids
Source: Nat Commun. 2018 Jan 16;9:234. doi: 10.1038/s41467-017-02742-3 (PMC5770463; doi:10.1038/s41467-017-02742-3)
Supplement: Supplementary file 1 — Supplementary Information [file 41467_2017_2742_MOESM1_ESM.pdf]

Supplementary Discussion

On the complexity of the kinetics of L-Phenylalanine addition to the R(FR)$_3$/(FE)$_4$ amyloid

To better characterize the reaction, we measured the early kinetics of the reaction for both **R(FR)$_3$/(FE)$_4$** and the soluble **R(FR)$_3$** at various L- and D-phenylalanine concentrations (Supplementary Figs. 6-8). In contrast to the additions of L- or D-phenylalanine to soluble peptide the initial rate of L-additions to **(FR)$_4$/(FE)$_4$** did not appear to be 1st order with respect to phenylalanine (Supplementary Fig. 8a). This finding indicates a complex mechanism, which is not unexpected considering that the observed concentration dependence of the stereoselectivty implies some binding interaction between the substrates before the reaction occurs (Fig. 2). It is reasonable to expect that the stability of the amyloid would inhibit the exchange of peptides on the time scale of the reaction and therefore preclude a truly catalytic mechanism. However, assuming a turn-over of 1, the initial rate of the amyloid reaction could be expected to follow Michaelis-Menten kinetics. We attempted to fit the initial rates to a Michaelis-Menten model but this also yielded only poor fits. Furthermore, the **(FR)$_4$/(FE)$_4$** reaction with L- but not D-phenylalanine appears to have a burst phase in the first few minutes of reaction, clearly visible in the 25, 100 and 200 μM reactions but absent in the D-additions of similar initial rate (at 1000, 2000 and 5000 μM D-phenylalanine, Supplementary Fig. 7). This is unlikely to be analogous to an enzyme burst rate because multiple turnover is not expected. Thus, no model for the L-phenylalanine additions to **(FR)$_4$/(FE)$_4$** can be put forward yet. This uncertainty is due to a number of factors: (i) The initial rate at 500 μM phenylalanine and above was too fast to be accurately measured. (ii) The activation of the amino acid involves at least one long-lived intermediate before the formation of the more reactive species (most likely N-carboxyanhydride)[1], and the concentrations of these species and the kinetics of their interconversion. (iii) The amyloid may structurally rearrange upon reaction, both locally by stabilizing the β-sheet (potentially influencing the neighboring reactive sites) as well as on the mesoscopic scale by enhancing protofilament-protofilament interactions (altering the accessibility of active sites). (iv) Polymorphisms may be present as often observed for amyloids[2] and these polymorphisms may exhibit distinct activities. In summary, while the kinetic data remain inconclusive concerning a model for the reaction mechanism, it does point to different mechanisms for the L-phenylalanine versus D-phenylalanine additions to the amyloid and all additions to the soluble peptide. Further studies, including high-resolution structure analyses of both the starting amyloid and product, are needed to fully explain the observed reaction kinetics.

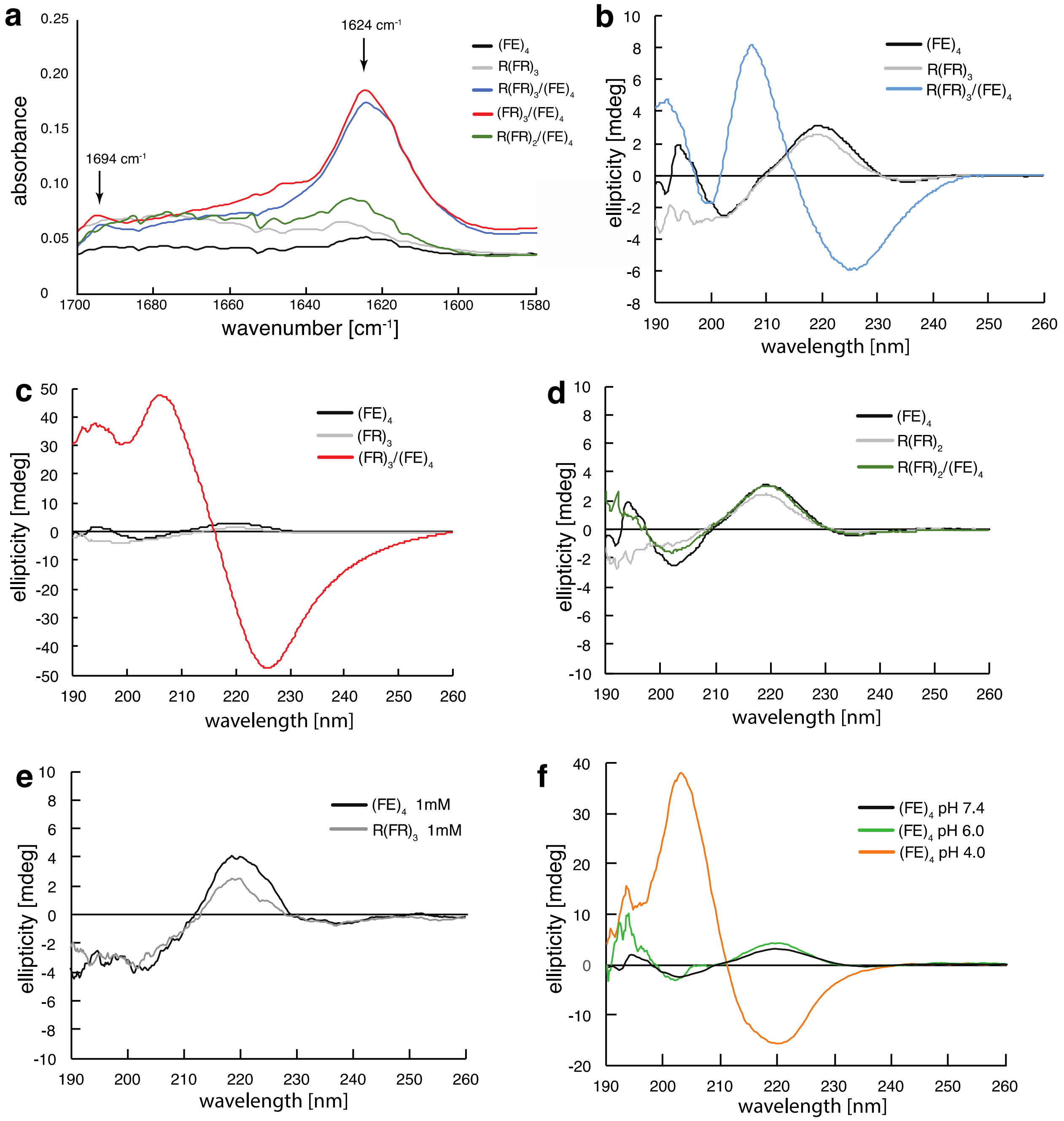

Supplementary Figure 1. Biophysical characterization of FR/FE peptides and their mixtures. Secondary structural details of peptide substrates, templates and their mixtures probed by Fourier Transform Infrared spectroscopy (**a**), and circular dichroism spectroscopy (**b,c,d**), The concentration of each peptide in **a-d** is 100 μM (in 20 mM buffer). To demonstrate their solubility, the spectra of the individual **(FE)$_4$** and **R(FR)$_3$** peptides were also measured at 1 mM (in 200 mM buffer) in a 0.1 mm path-length cuvette (**e**) yielding nearly identical spectra to those in **b**. At 100 μM, **(FE)$_4$** displays a pH dependent conversion to beta structure (**f** and Supplementary Fig. 2e). The sample at pH 4 was in 20 mM NaOAc. For pH 6 and 7.4, 20 mM $NaPO_4$ was used. Peptides with sequence composition (FE)$_n$ have been previously shown to be surface active and prone to β-aggregation. They also have an isoelectric point several pH units above their calculated value. However, comparison of panels **a** and **e**, indicates that at pH 7.4, **(FE)$_4$** is soluble and not β-structured even at a concentration of 1 mM in 200 mM $NaPO_4$ pH 7.4, consistent with previous studies on a similar peptide[3].

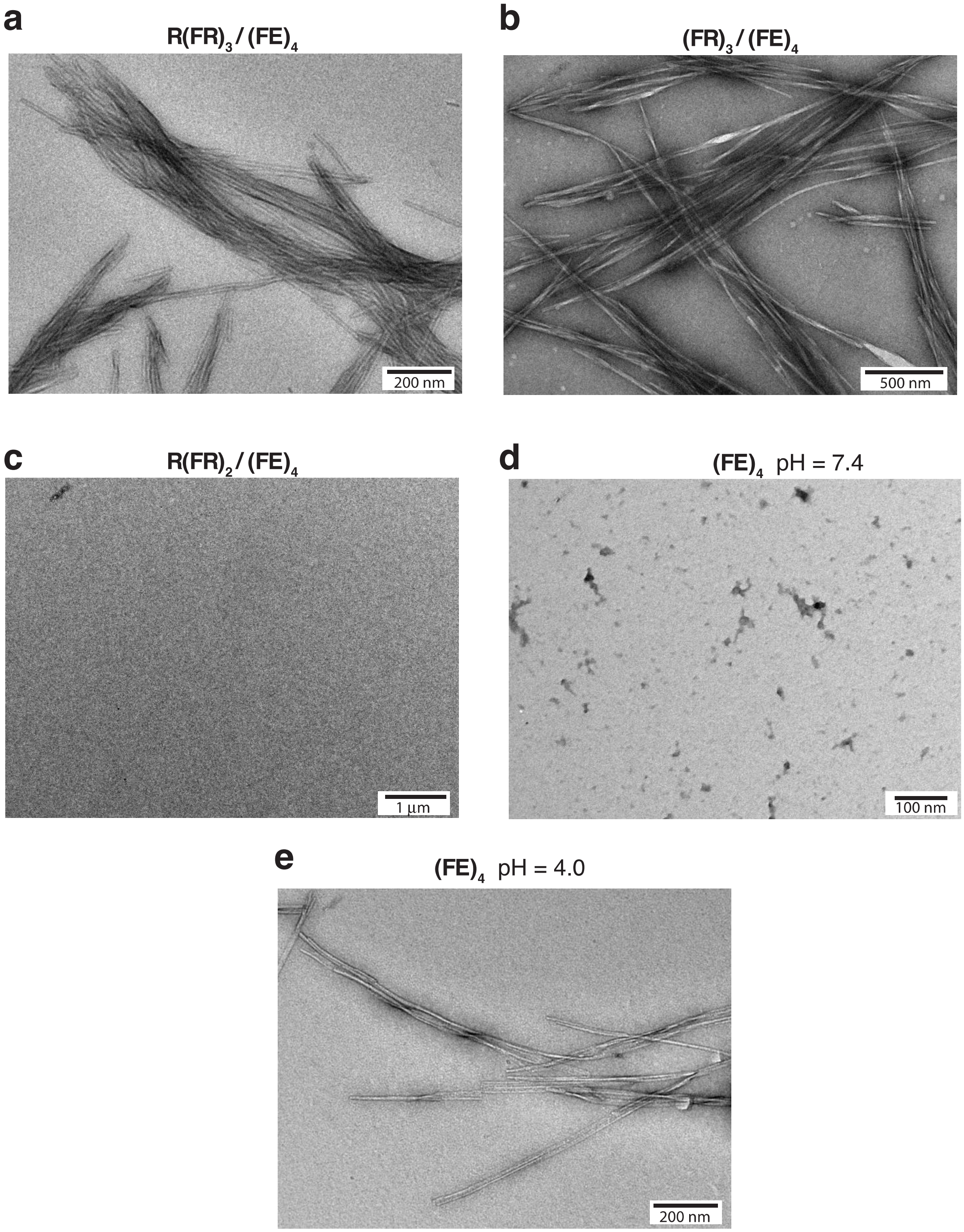

Supplementary Figure 2. Morphological characterization of FR/FE peptides and their mixtures. Electron micrographs of peptide mixtures showing amyloid like structures in transmission electron micrographs (**a,b**). The **$R(FR)_2(FE)_4$** mixture does not display any aggregate by EM (**c**). The **$(FE)_4$** peptide alone at pH 7.4 yielded mostly empty regions with some amorphous aggregates (**d**). However, below its pI, at pH 4 **$(FE)_4$** does form amyloids on its own (**e**). The binary peptide mixtures for electron microscopy were 100 μM each peptide. The **$(FE)_4$** sample at pH 7.4 was at 450 μM (20mM $NaPO_4$) and at pH 4 it was 200 μM (20 mM NaOAc).

Figure S3 (legend on following page)

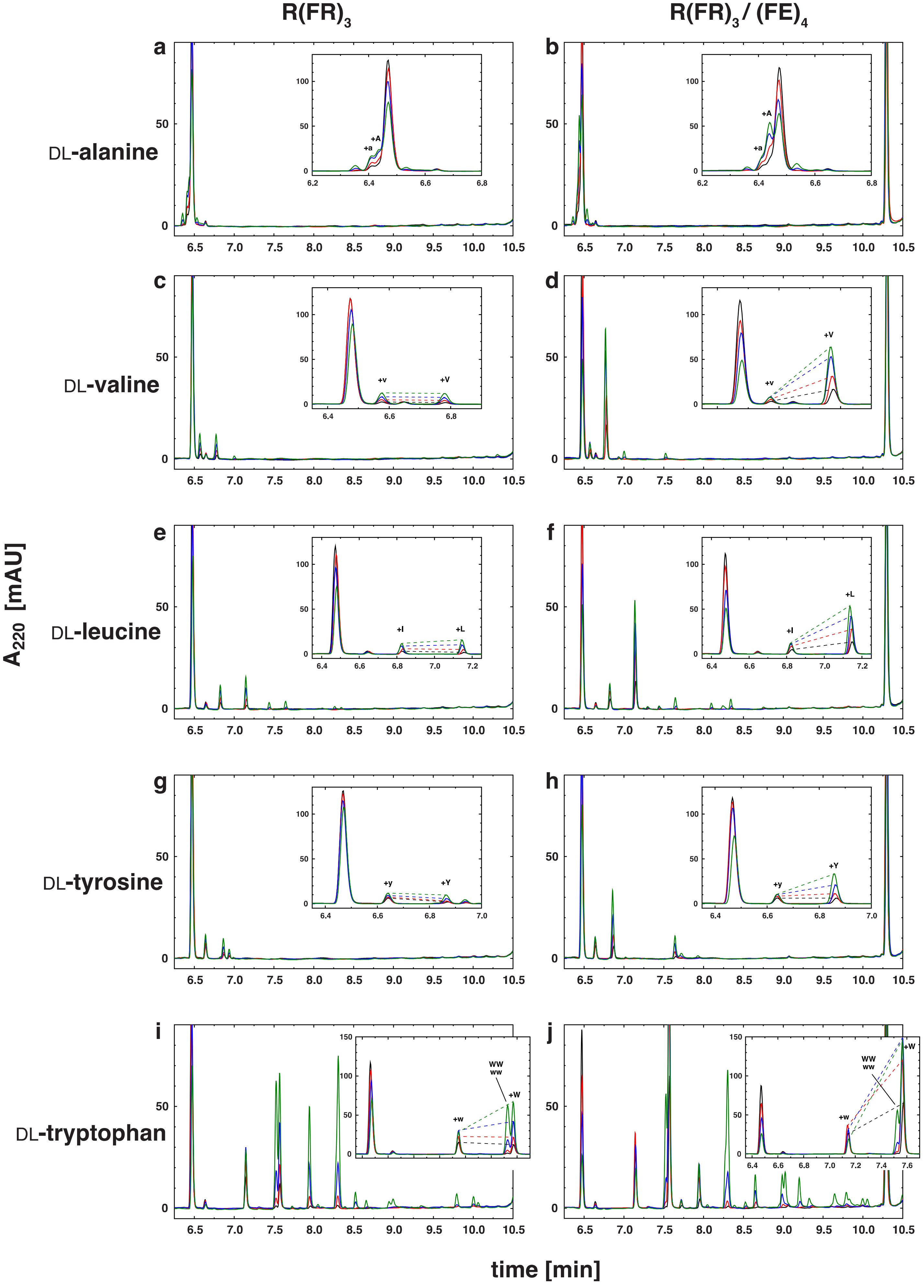

Supplementary Figure 3. Stereoselective addition reactions with the R(FR)$_3$/(FE)$_4$ amyloid. Reverse phase HPLC chromatograms of the addition reactions of DL-alanine, DL-valine, DL-leucine, DL-tyrosine and DL-tryptophan with soluble substrate **R(FR)$_3$** (a, c, e, g, i) and with amyloid **R(FR)$_3$/(FE)$_4$** (b, d, f, h, j). The **R(FR)$_3$** concentration was 100 μM, the **(FE)$_4$** concentration was 130 μM and amino acids were at concentrations of 25 μM (black), 50 μM (red), 100 μM (blue), 200 μM (green) of each enantiomer. The inset plot represents the zoomed-in region around the two enantiomeric single addition products. The addition products for the D- and L- enantiomers are indicated in lower and upper case letters, respectively. The single addiction product diastereomers are connected with a dashed line to illustrate differences in the yield of the products at different concentration of amino acid. The product enantiomeric excesses are tabulated in Supplementary Table 2.

Supplementary Fig. 4 (page 1 of 3)

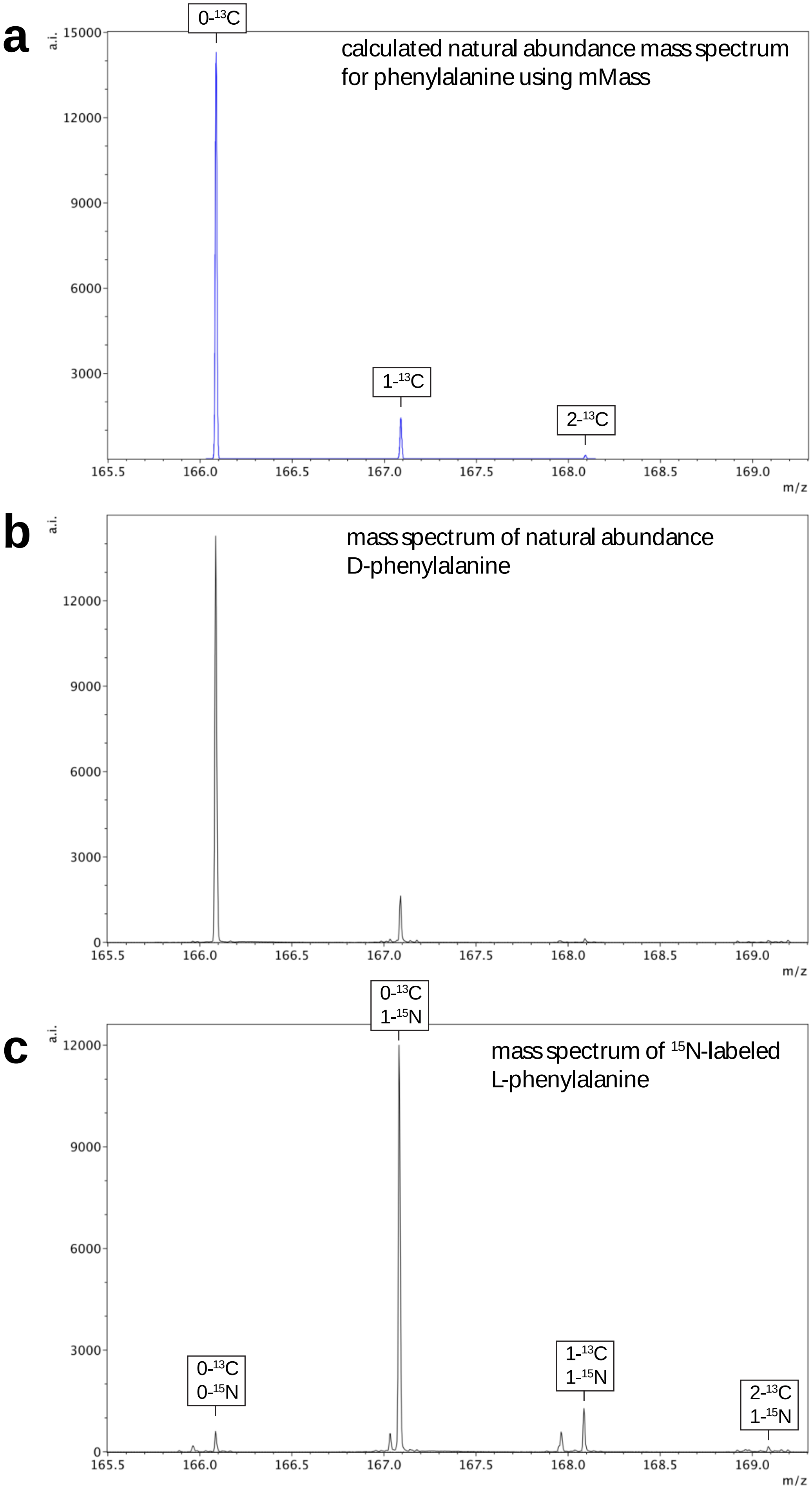

d

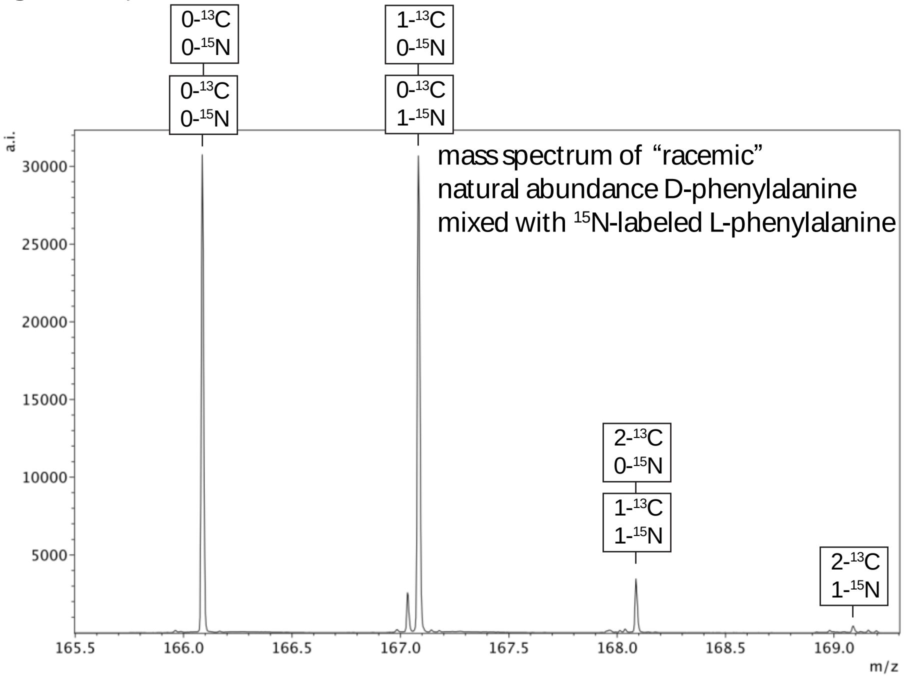
0-^{13}C
0-^{15}N
0-^{13}C
0-^{15}N
1-^{13}C
0-^{15}N
0-^{13}C
1-^{15}N
2-^{13}C
0-^{15}N
1-^{13}C
1-^{15}N
2-^{13}C
1-^{15}N
a.i.
30000
25000
20000
15000
10000
5000
165.5
166.0
166.5
167.0
167.5
168.0
168.5
169.0
m/z
mass spectrum of "racemic" natural abundance D-phenylalanine mixed with ^{15}N-labeled L-phenylalanine

e

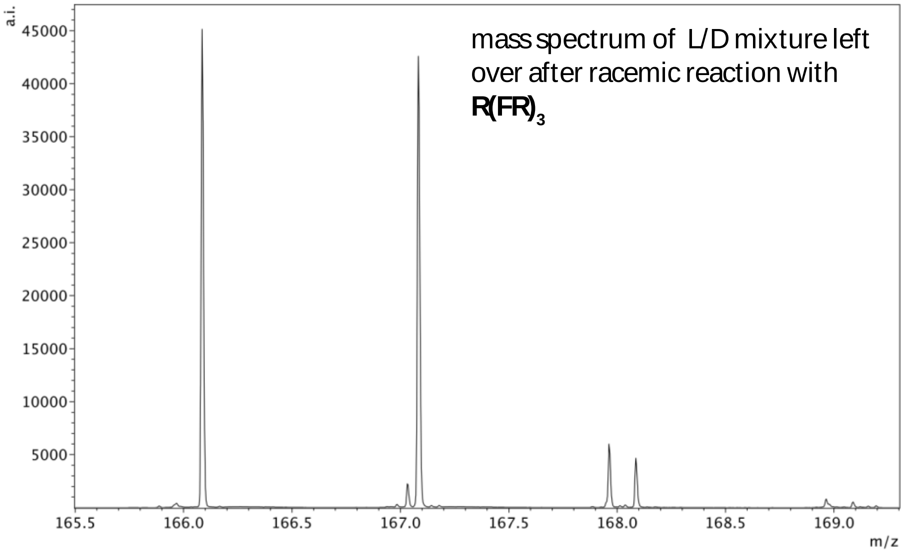
a.i.
45000
40000
35000
30000
25000
20000
15000
10000
5000
165.5
166.0
166.5
167.0
167.5
168.0
168.5
169.0
m/z
mass spectrum of L/D mixture left over after racemic reaction with **R(FR)$_3$**

f

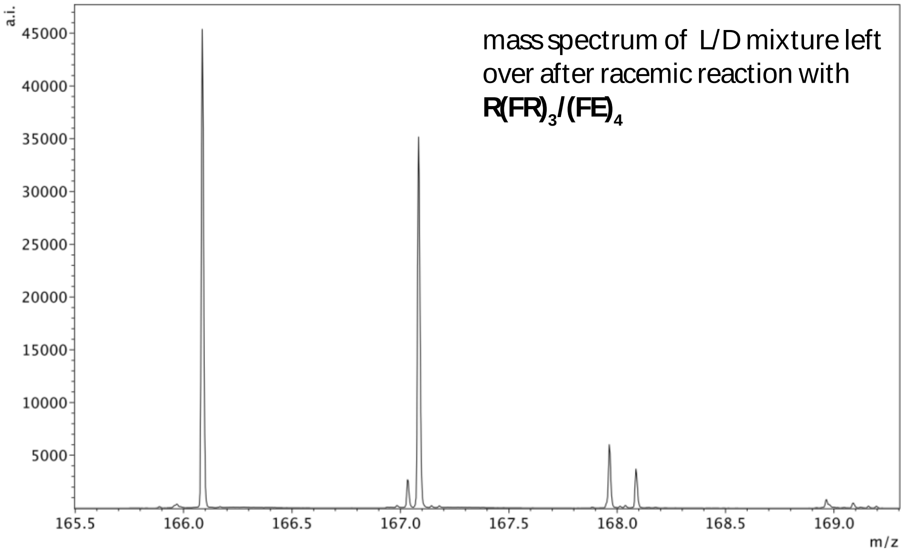
a.i.
45000
40000
35000
30000
25000
20000
15000
10000
5000
165.5
166.0
166.5
167.0
167.5
168.0
168.5
169.0
m/z
mass spectrum of L/D mixture left over after racemic reaction with **R(FR)$_3$/(FE)$_4$**

Supplementary Figure 4. Mass spectral analysis of the enantiomeric composition of non-reacted phenylalanine. The standard reaction with 100 μM **R(FR)₃** or 100 μM **R(FR)₃/(FE)₄**) and a quasi-racemic mixture of 100 μM each ^{15}N-labeled L-phenylalanine and natural abundance D-phenylalanine was allowed to reach completion (24h). The reactions were analyzed by HPLC, the phenylalanine peaks collected, dried under vacuum and resuspended in 50% CH3CN, 0.1% formic acid. Controls of L- and D-phenylalanine and their quasi-racemic mixture were similarly HPLC purified and prepared for MS. The phenylalanine samples were analyzed by ESI-QTOF mass spectrometry on a maXis instrument (Bruker) and the peak areas quantitated with the mMass[4] software. Peak integration was more accurate than peak height for quantitation because the maXis does not resolve the ^{15}N and ^{13}C isotopes. However, their peaks positions are slightly different and so the overlap of their peaks is not perfect, resulting in a slightly broader peak whose area, but not height, is representative of the total signal. The calculated spectrum for natural abundance phenylalanine (**a**) very closely matches the real D-phenylalanine spectrum (**b**). The spectrum of the ^{15}N-labeled L-phenylalanine (**c**) has the expected pattern including the small peak at 166.086 Da resulting from the fact that the ^{15}N labeling is only ~98%. The peak areas of the remaining three spectra (**c-d**) were fit to a linear combination of the peak area ratios in a and b (all to within 0.5% difference). The quasi-racemic mixture (**c**) is best fit to a mixture of 48.8% D, 51.2% L (2.4% ee). The leftover phenylalanine in the **R(FR)₃** reaction (**d**) is 50.7% D, 49.3% L (-1.4% ee) and the leftover phenylalanine in the **R(FR)₃/(FE)₄** reaction (**e**) is 56.8% D, 43.2% L (-13.6% ee). The **R(FR)₃** reaction from which the leftover phenylalanine in d was recovered had a final concentration of 5 μM D-addition product and 8 μM L-addition product. The **R(FR)₃/(FE)₄** reaction from which the leftover phenylalanine in e was recovered had a final concentration of 2 μM D-addition product and 22 μM L-addition product. Considering that the starting concentration was 100 μM of each enantiomer, the remaining phenylalanine e.e. (mass spec results) are consistent with the observed product d.e. (HPLC peak areas).

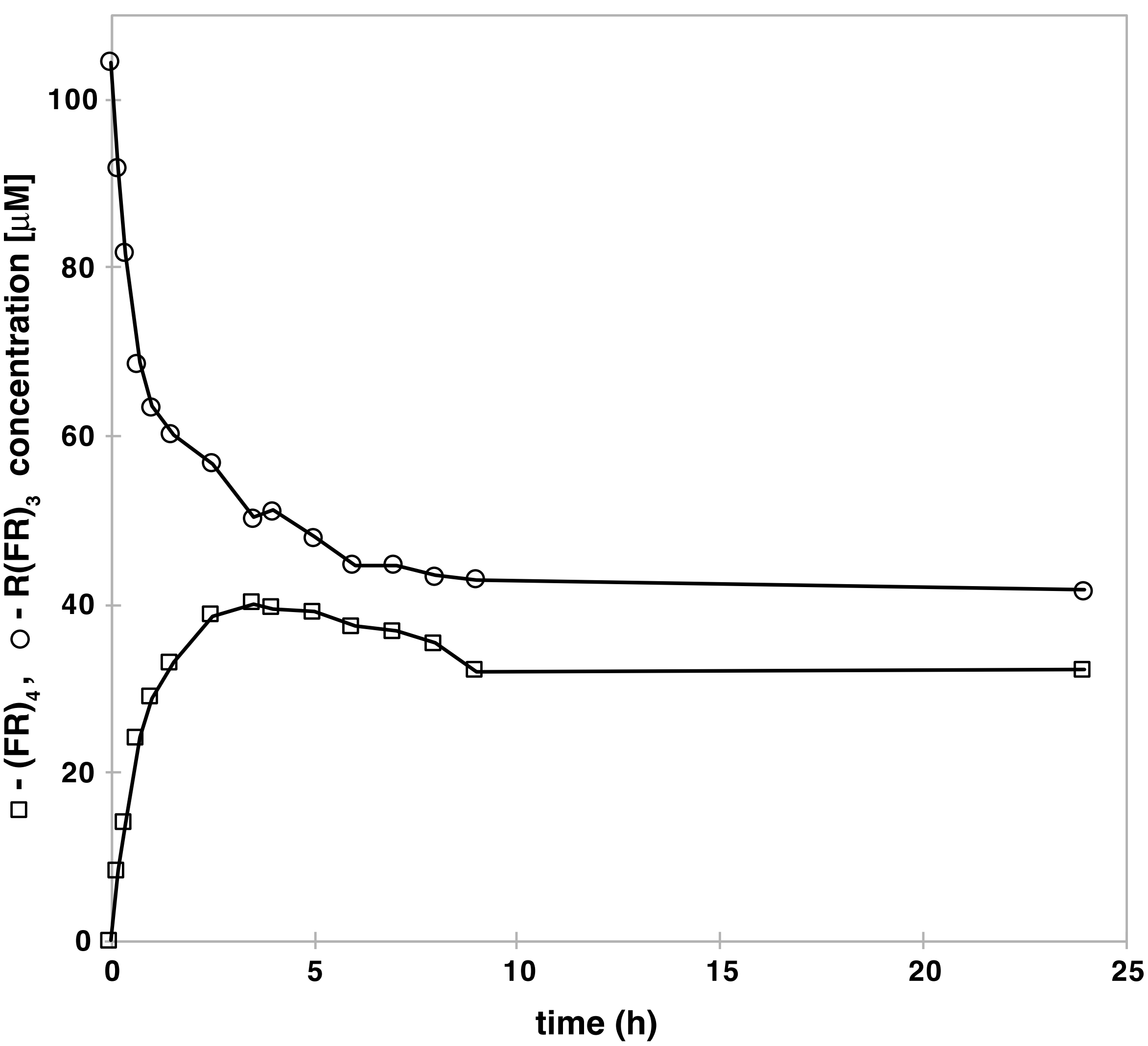

Supplementary Figure 5. Time dependence of $(FR)_4$ and $R(FR)_3$ concentrations upon addition of activated phenylalanine to $R(FR)_3/(FR)_4$. The standard reaction conditions with 100 μM of each peptide and 100 μM L-phenylalanine were carried out with a larger volume so that aliquots could be removed at time points and quenched by 4-fold dilution into 8 M guanidine with 45 mM HCl. The aliquots were analyzed by HPLC and the concentration of the starting peptide (circles) and the addition product (squares) were quantitated by their peak areas. The data show that the majority of the **$(FR)_4$** product is formed within 30 min, with the yield peaking around 3 hours and then sinking by about 20%, primarily due to multiple additions.

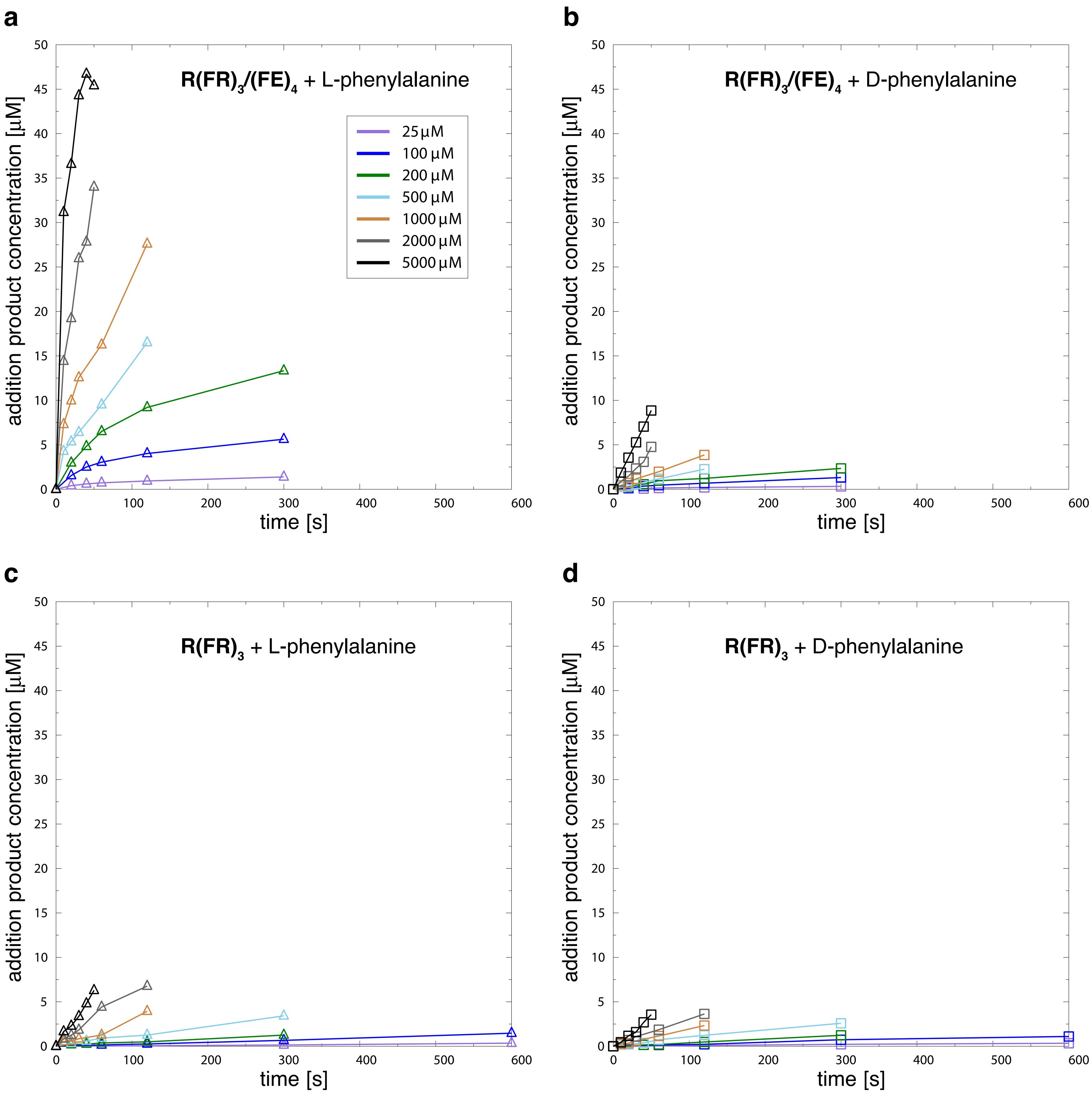

Supplementary Figure 6. Early kinetics of phenylalanine addition. The initial rate of L- or D-phenylalanine addition to **$R(FR)_3/(FR)_4$** (**a**,**b**) and **$R(FR)_3$** (**c**,**d**) are plotted on a single scale (individually scaled plots are shown in Fig. S8). The product concentration was determined by HPLC (based on the peak areas of the reverse phase chromatogram) for five time points for each phenylalanine concentration. The legend in (**a**) applies to all four panels. The triangles are for L additions and the squares are for D additions. The faster kinetics of the amyloid substrate is evident as well as the preference for L-phenylalanine.

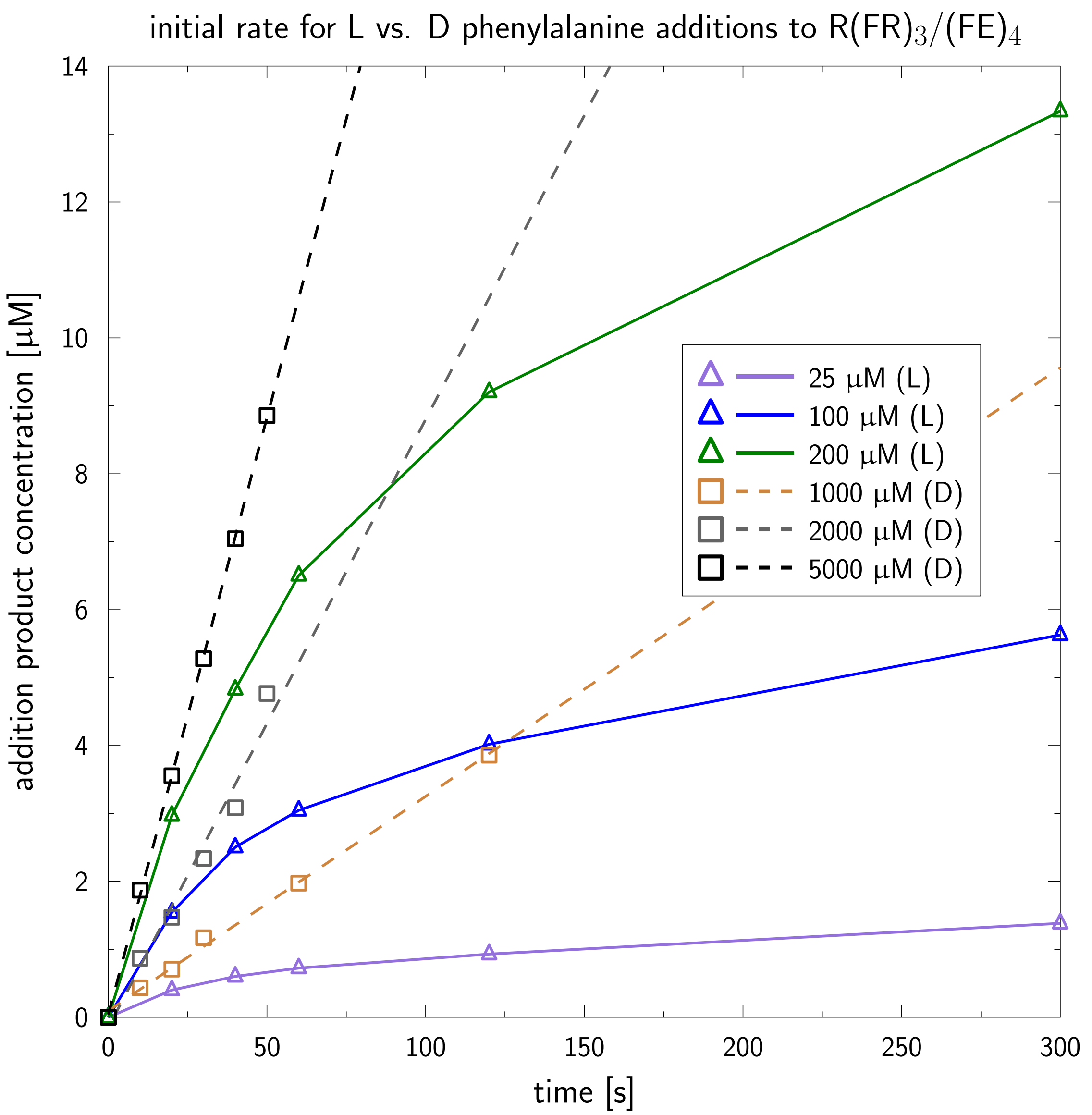

Supplementary Figure 7. Comparison of the early kinetics of L versus D additions to R(FR)$_3$/(FR)$_4$. The three lowest concentrations from Supplementary Fig. 6a and the three highest from Supplementary Fig. 6b are plotted together to highlight the different evolution of the rate: it remains constant for D additions (squares) but changes rapidly for L additions (triangles). The solid lines are a fit to the five time points (and t = 0 s), while the dashed lines simply connect one data point to the next. Note: all of the D–phenylalanine addition reactions and the reactions with soluble **R(FR)$_3$** had initial rates that fit well to a line. This data in this figure was selected simply to better compare reactions that had similar rates and similar concentrations of product. This highlights the distinct reaction mechanism in the amyloid for homochiral L–phenylalanine addition compared with the heterochiral D–phenylalanine addition.

Supplementary Fig. 8 (figure legend on the following page)

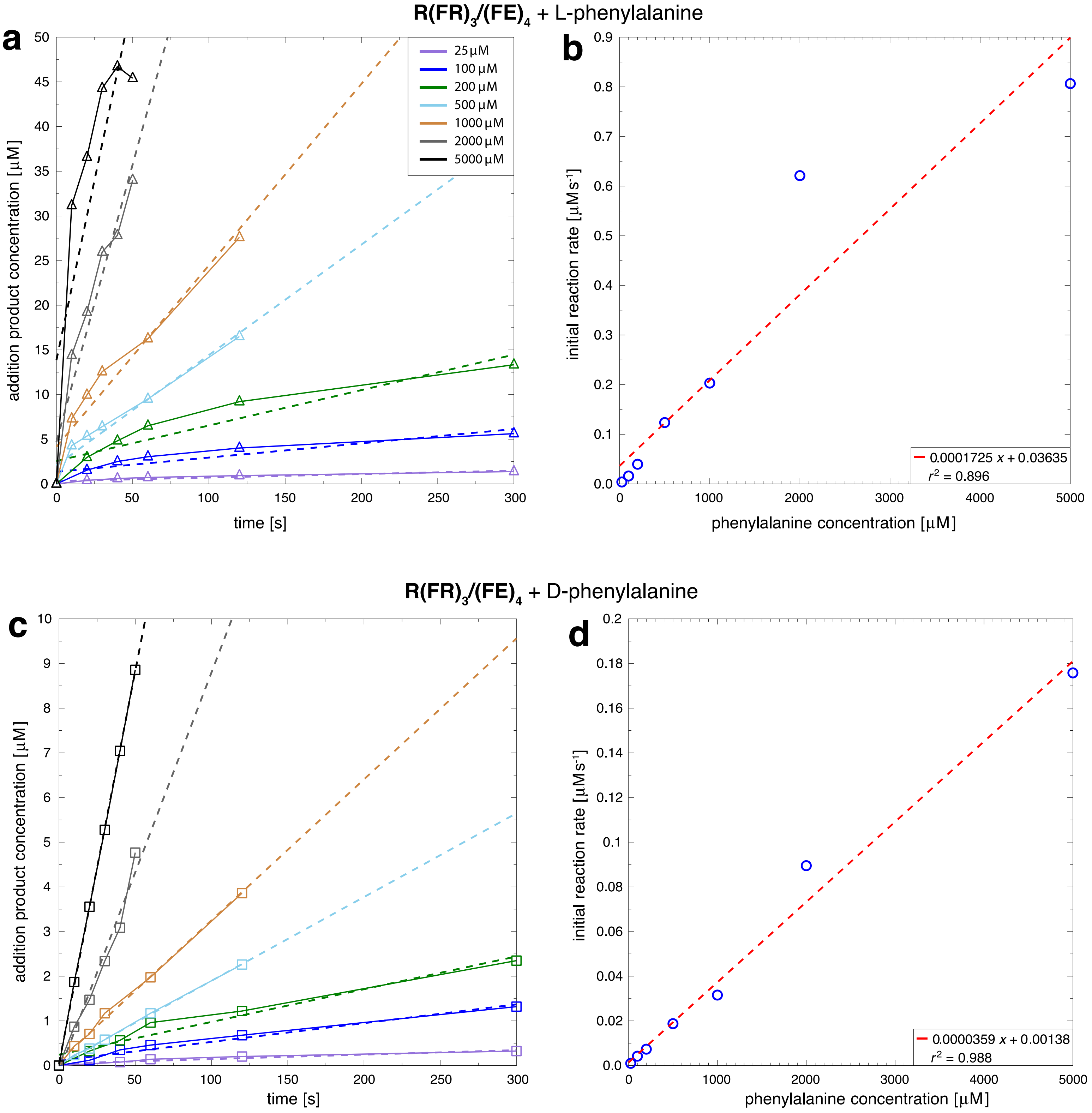

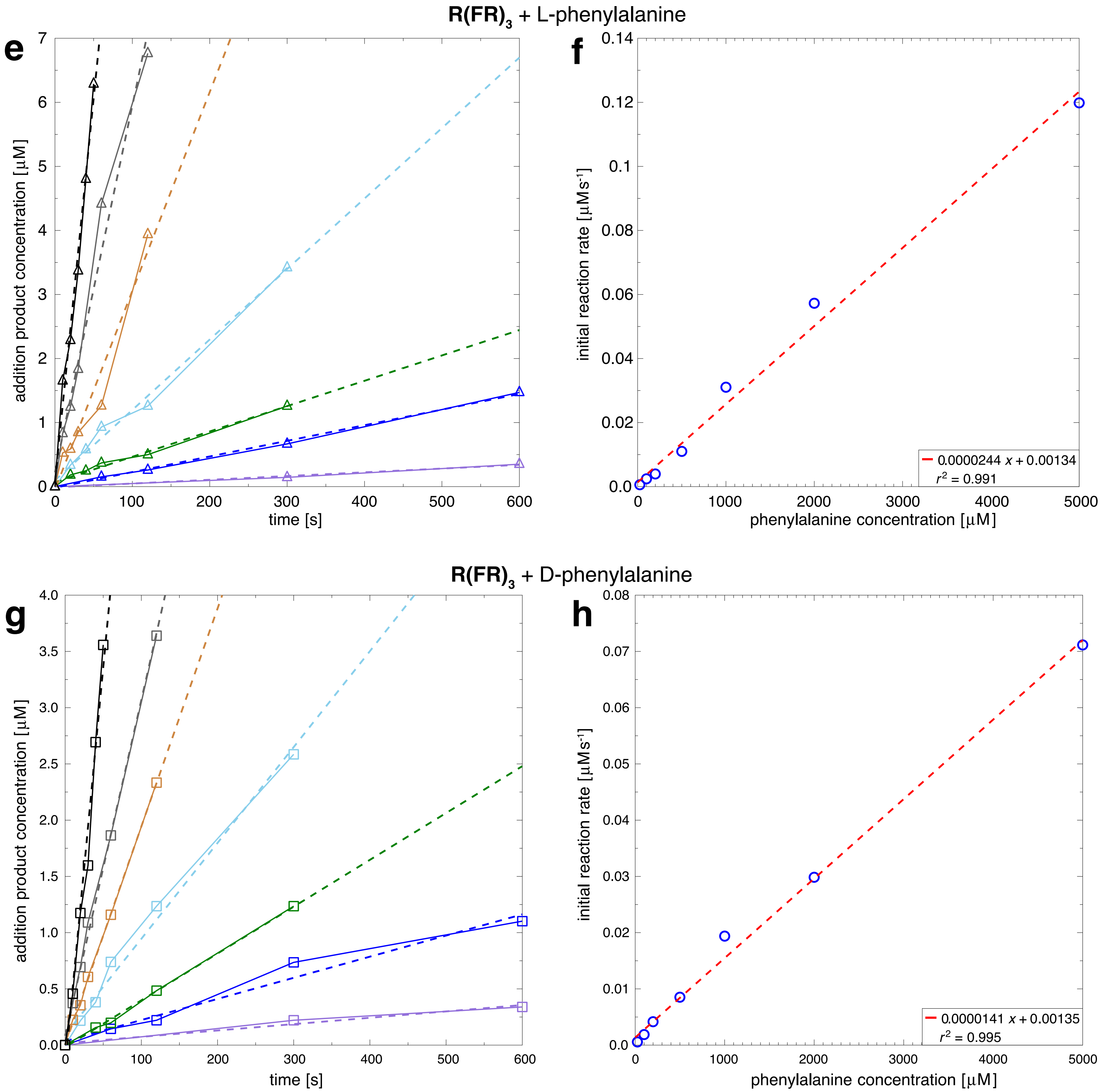

Supplementary Figure 8. Early kinetics and fit of initial rate to a 1st order mechanism. The data for each phenylalanine concentration depicted in Supplementary Fig. 6 was fit to a line (a, c, e, g) and the slope of this line (initial rate) plotted against the phenylalanine concentration (b, d, f, h). The dashed lines are the fits and the solid lines connect the data points. The color scheme for all plots is as in **a**. The data in **a** is clearly not indicative of a simple reaction mechanism while the others are reasonably well described by a 1st order reaction (with respect to phenylalanine). The data in **a** is also very poorly fit by a Michealis Menten model (not shown), however this could be because the initial rates in **a** could not be properly measured (see discussion above).

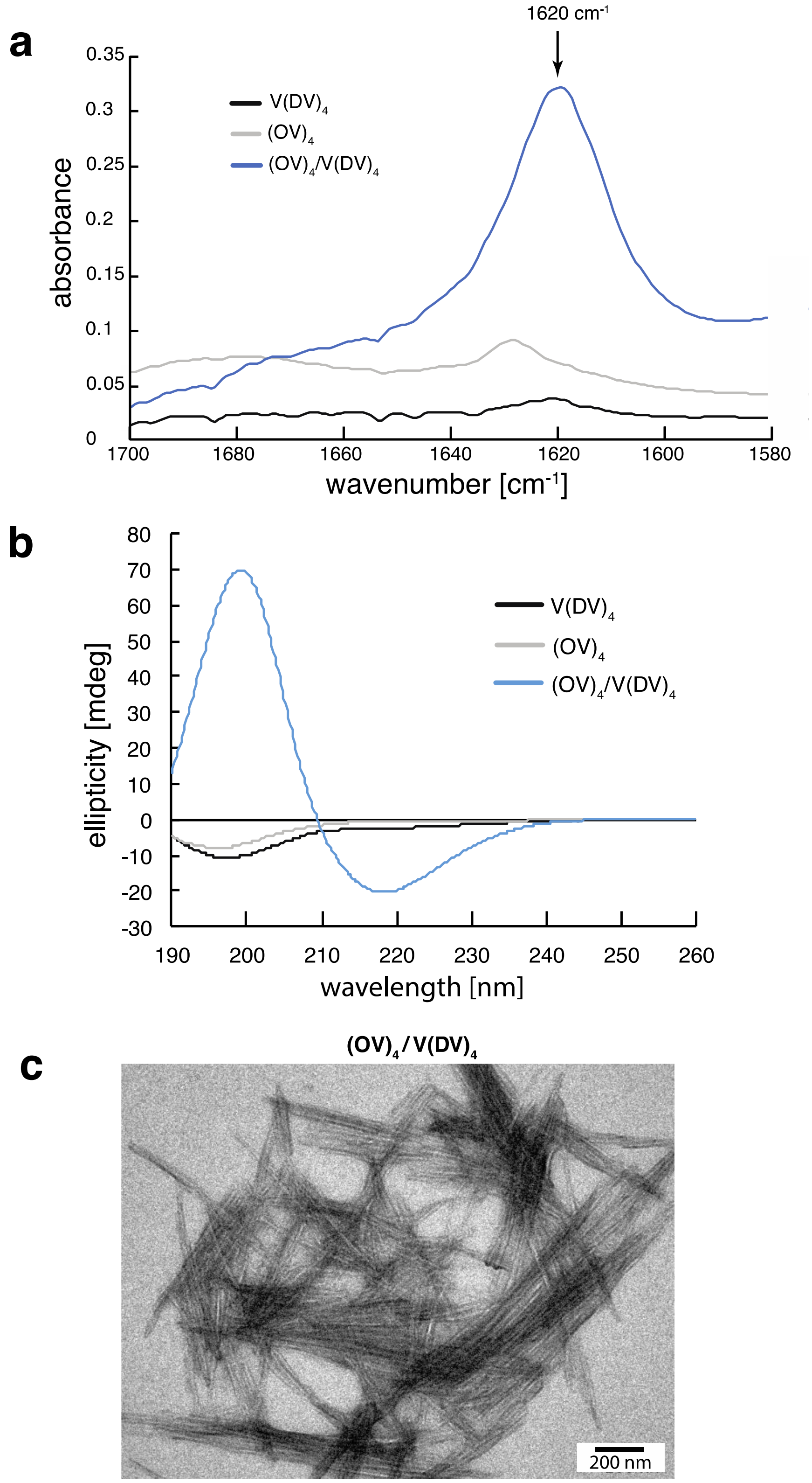

Supplementary Figure 9. Biophysical and morphological characterization of OV/VD peptides. Secondary structural details of peptide substrates, templates and their mixtures probed by Fourier Transform Infrared spectroscopy (**a**), circular dichroism spectroscopy (**b**), and morphological details of the **$(OV)_4/V(DV)_4$** peptide mixture showing amyloid like structures by transmission electron microscopy (**c**).

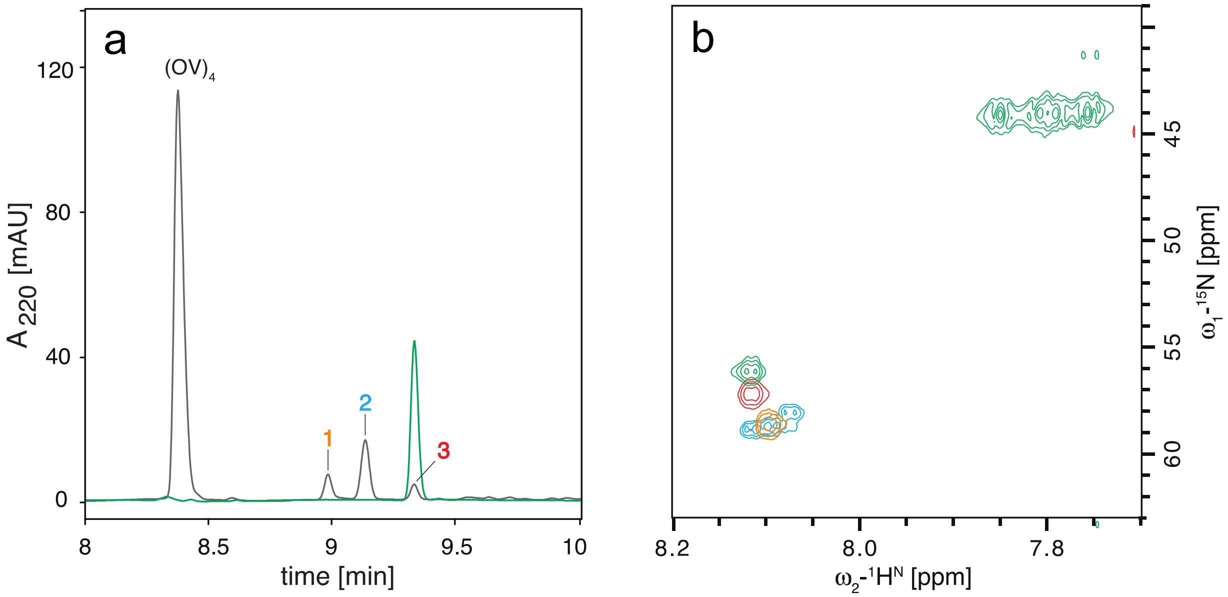

Supplementary Figure 10. Identification of N-terminal valine addition product of (OV)$_4$ peptide. Using an authentic **V(OV)$_4$** peptide, the elution time of the N-terminal addition product and the identity of the four other sidechain addition products were confirmed by their retention time on reverse phase HPLC (**a**), and via 2-D HMQC NMR spectroscopy (**b**). The control peptide (green trace in **a**) co-elutes with peak 3. The natural abundance spectrum of the authentic **V(OV)$_4$** is in green contours and shows the 4 triplets for the side-chain amines and the doublet for the N-terminal amine. HPLC peaks 1, 2, and 3 in **a** were purified from a reaction of ^{15}N-isotope enriched valine with natural abundance **(OV)$_4$**. Their HMQC spectra (only ^{15}N-enriched valine detected) are shown in **b** and colored to match the peak labels in **a**. Peak 1 is a single product, peak 2 has three products, and peak 3 is the N-terminal addition product.

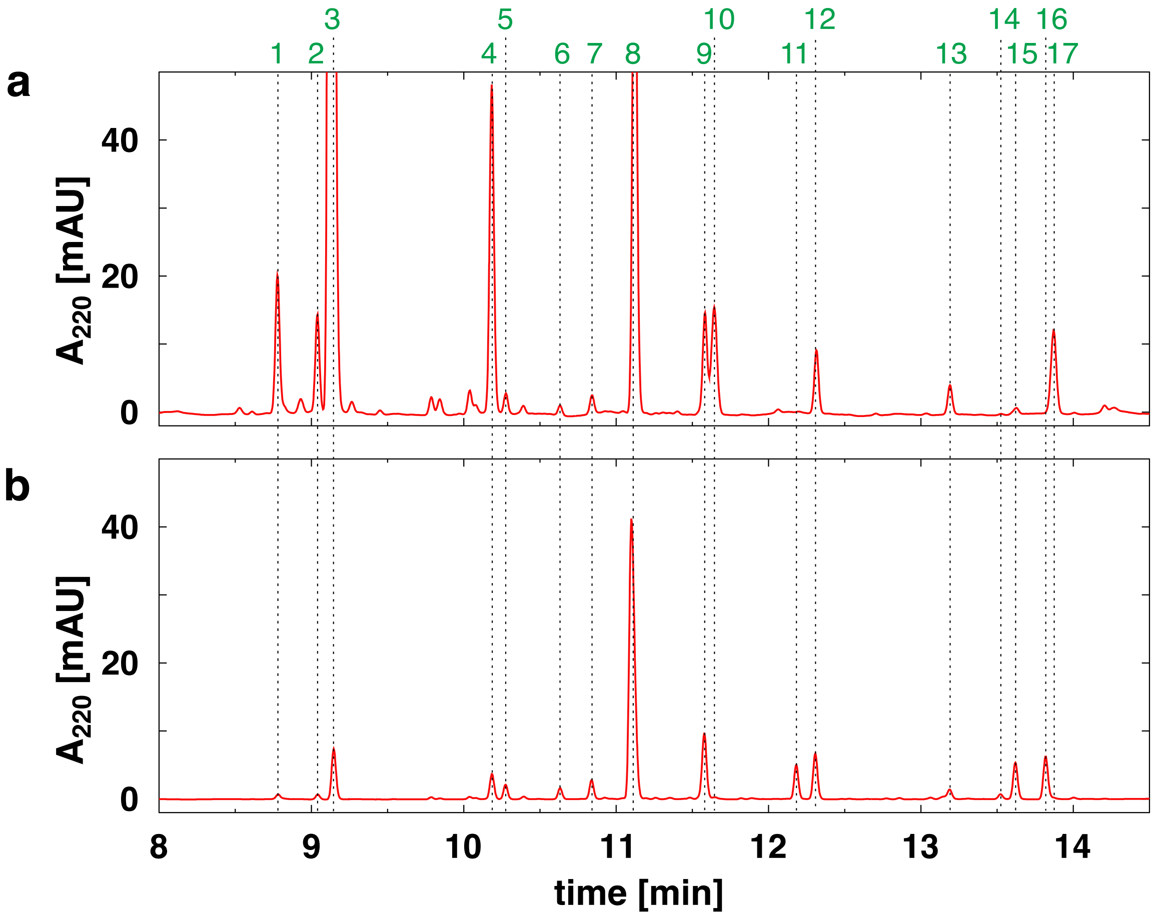

a
b
1 2 3 4 5 6 7 8 9 10 11 12 13 14 15 16 17
A220 [mAU]
time [min]

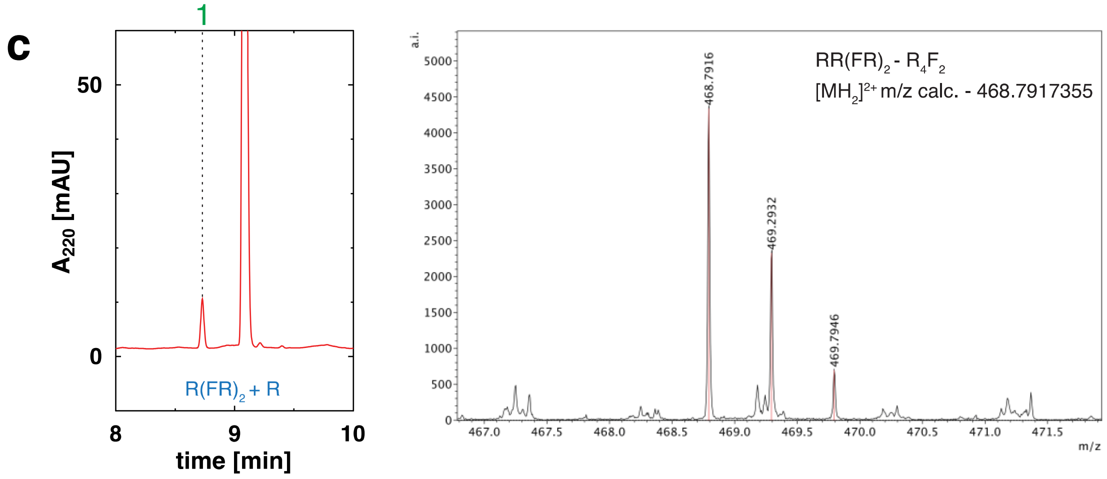

c
1
A220 [mAU]
time [min]
R(FR)2 + R
RR(FR)2 - R4F2
[MH2]2+ m/z calc. - 468.7917355
468.7916
469.2932
469.7946
a.i.
m/z

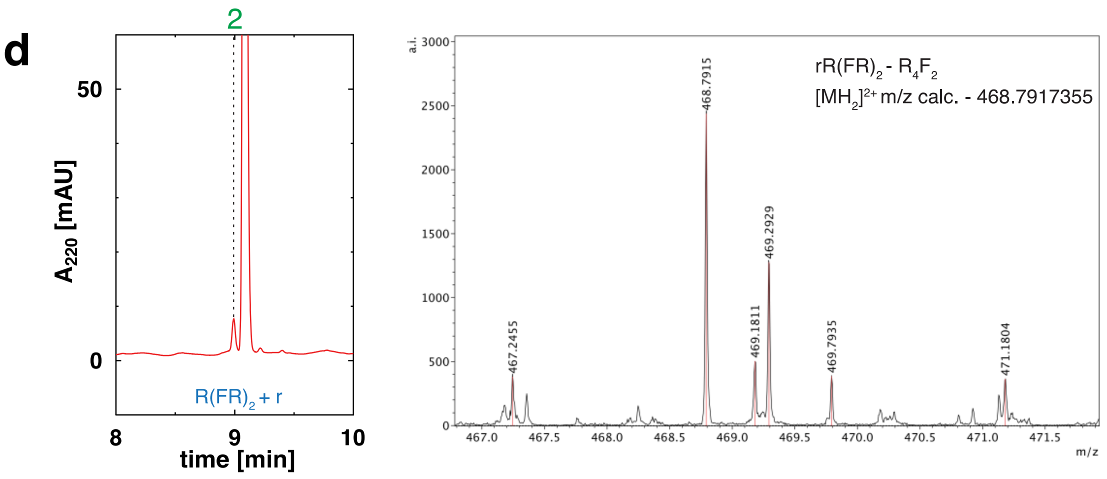

d
2
A220 [mAU]
time [min]
R(FR)2 + r
rR(FR)2 - R4F2
[MH2]2+ m/z calc. - 468.7917355
467.2455
468.7915
469.1811
469.2929
469.7935
471.1804
a.i.
m/z

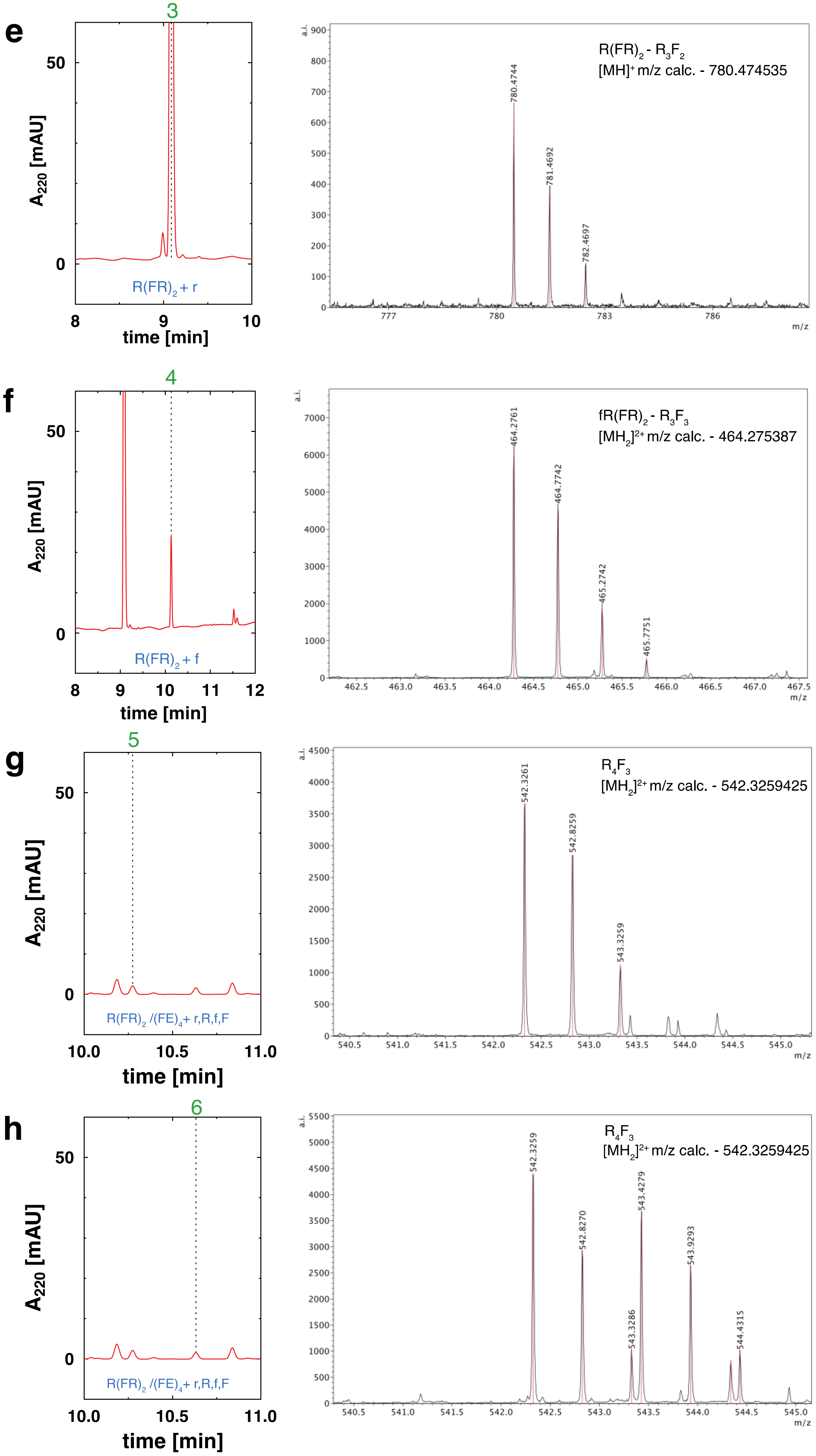
e
3
A220 [mAU]
50
0
R(FR)2 + r
8
9
10
time [min]
R(FR)2 - R3F2
[MH]+ m/z calc. - 780.474535
780.4744
781.4692
782.4697
a.i.
m/z
f
4
R(FR)2 + f
11
12
fR(FR)2 - R3F3
[MH2]2+ m/z calc. - 464.275387
464.2761
464.7742
465.2742
465.7751
g
5
R(FR)2 /(FE)4+ r,R,f,F
10.0
10.5
11.0
R4F3
[MH2]2+ m/z calc. - 542.3259425
542.3261
542.8259
543.3259
h
6
R(FR)2 /(FE)4+ r,R,f,F
R4F3
[MH2]2+ m/z calc. - 542.3259425
542.3259
542.8270
543.3286
543.4279
543.9293
544.4315

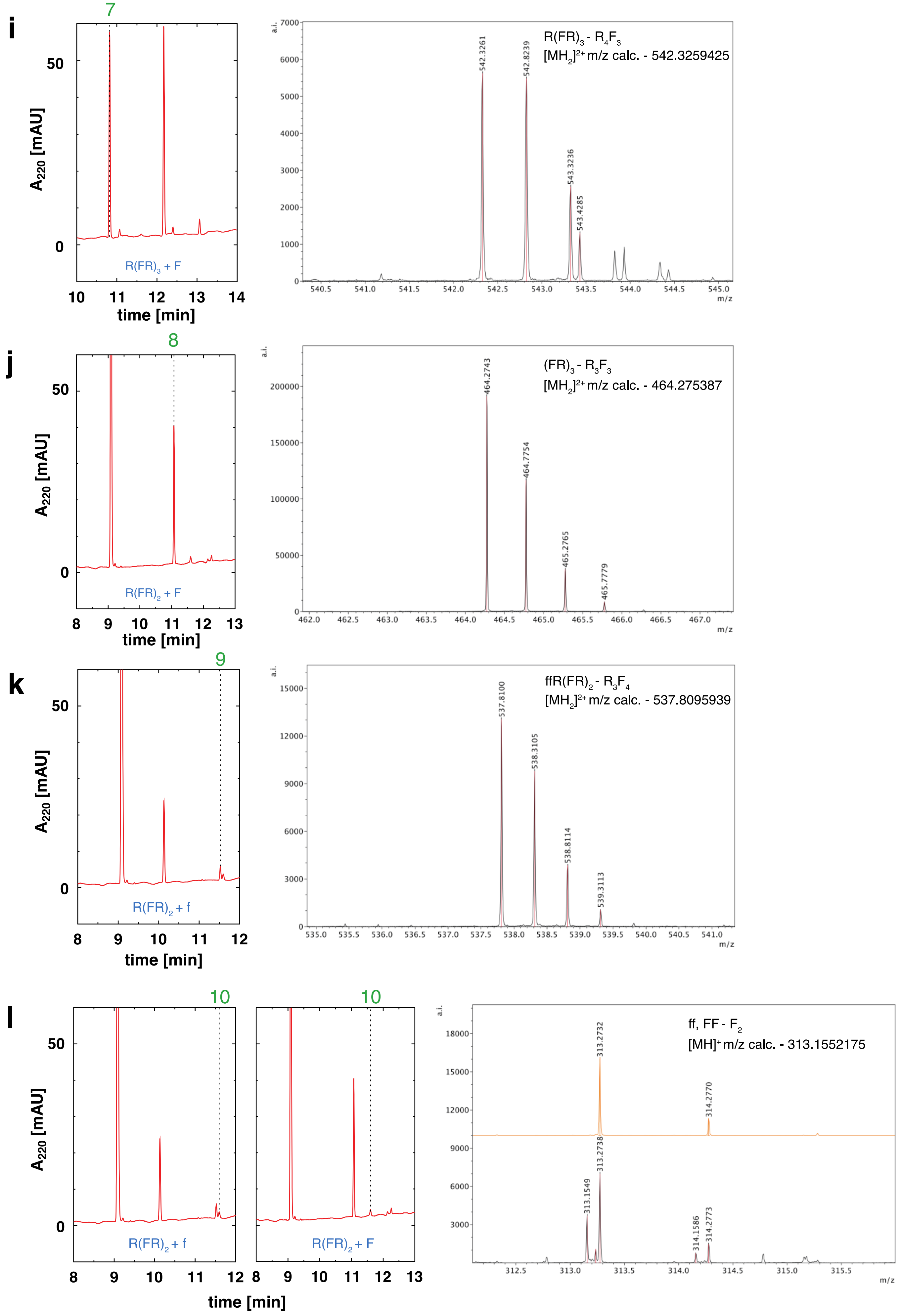
i
7
A220 [mAU]
R(FR)3 + F
time [min]
R(FR)3 - R4F3
[MH2]2+ m/z calc. - 542.3259425
542.3261
542.8239
543.3236
543.4285
j
8
R(FR)2 + F
(FR)3 - R3F3
[MH2]2+ m/z calc. - 464.275387
464.2743
464.7754
465.2765
465.7779
k
9
R(FR)2 + f
ffR(FR)2 - R3F4
[MH2]2+ m/z calc. - 537.8095939
537.8100
538.3105
538.8114
539.3113
l
10
R(FR)2 + f
R(FR)2 + F
ff, FF - F2
[MH]+ m/z calc. - 313.1552175
313.2732
314.2770
313.2738
313.1549
314.1586
314.2773
a.i.
m/z

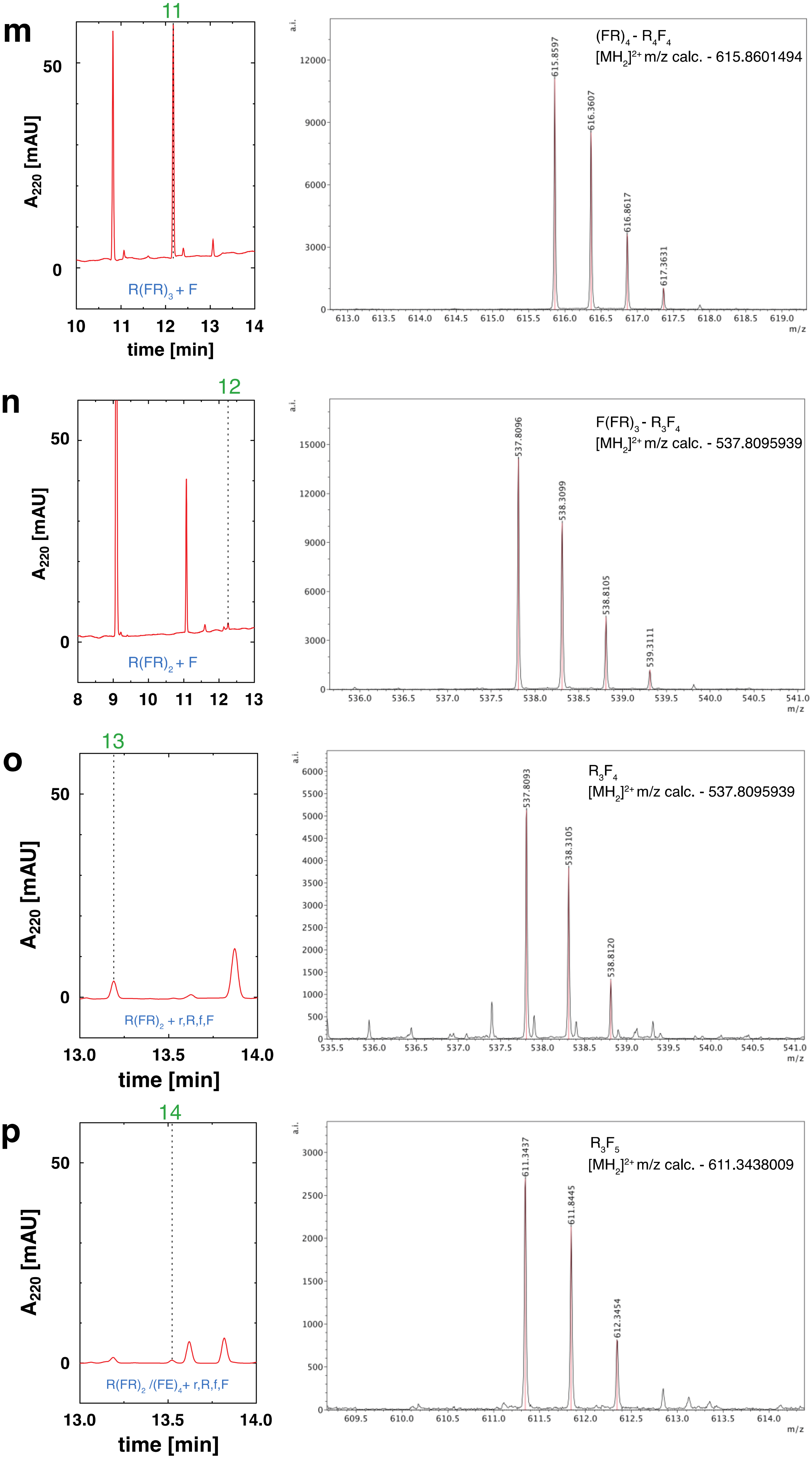

m
11
A220 [mAU]
R(FR)3 + F
time [min]
(FR)4 - R4F4
[MH2]2+ m/z calc. - 615.8601494
615.8597
616.3607
616.8617
617.3631
n
12
R(FR)2 + F
F(FR)3 - R3F4
[MH2]2+ m/z calc. - 537.8095939
537.8096
538.3099
538.8105
539.3111
o
13
R(FR)2 + r,R,f,F
R3F4
[MH2]2+ m/z calc. - 537.8095939
537.8093
538.3105
538.8120
p
14
R(FR)2 /(FE)4+ r,R,f,F
R3F5
[MH2]2+ m/z calc. - 611.3438009
611.3437
611.8445
612.3454
m/z
a.i.

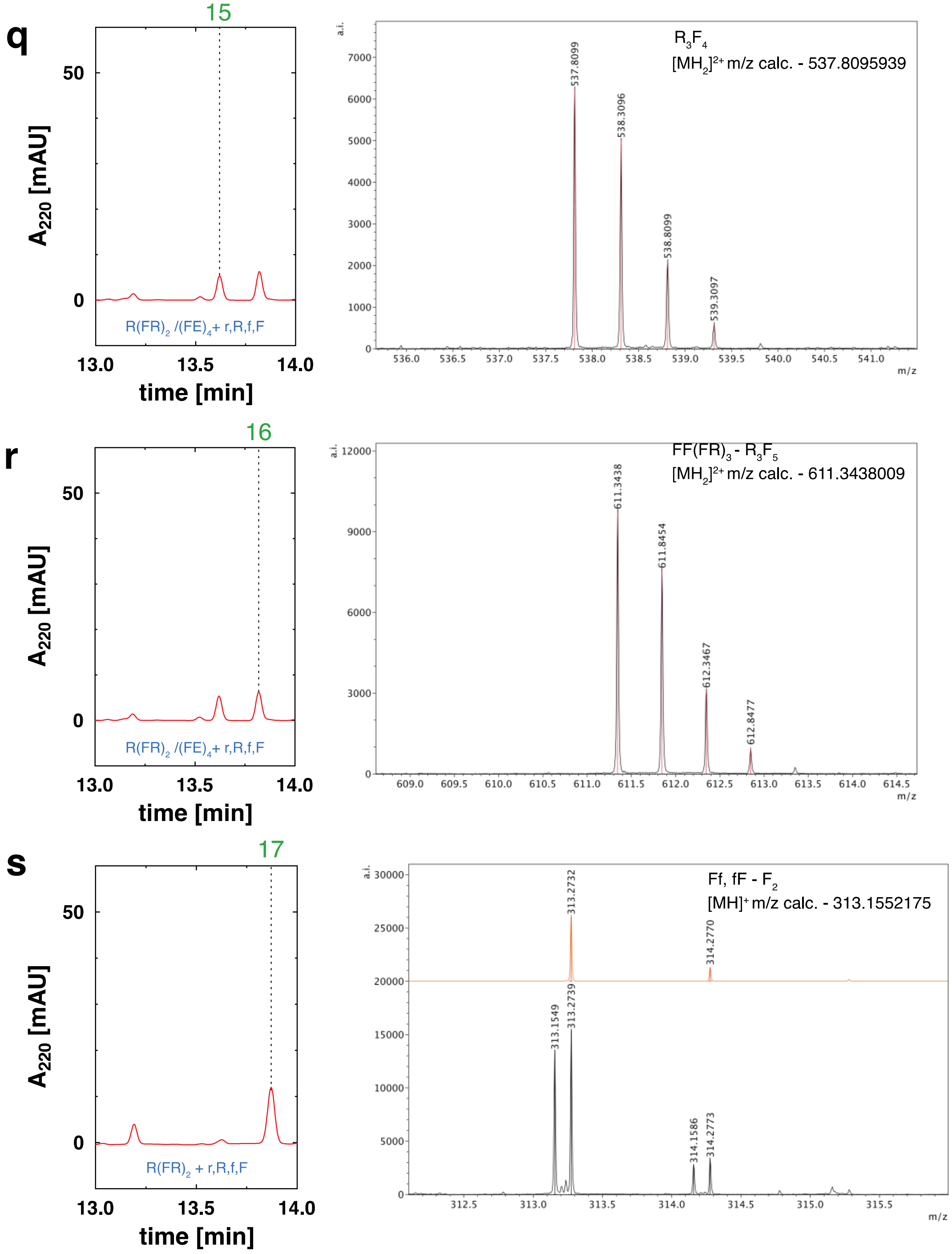

Supplementary Figure 11. Identification of the sequence/composition of peptides in Fig. 4. The identification of peaks in the HPLC chromatogram of Fig. 4 was performed by a combination of mass identification to assign the composition and when available, retention time of a reaction product with limited sequence possibilities. For example, a measured composition of R3F3 could be identified as fRFRFR if its retention time matched that of the product of the reaction of **R(FR)₂** + f but not to the retention time any products of **R(FR)₂** + F. This series of deductions is laid out in the chromatograms and mass spectra in panels c-s. All of the mass spectra are from samples collected during the HPLC analysis of either the supernatant fraction (a) or the insoluble fraction (b) of the **R(FR)₂/(FE)₄** reaction with a racemic mixture of phenylalanine and arginine (from Fig 4b). The chromatograms in c-s are from reactions described in blue text below their traces. For peptides for which only the composition could be identified, one of the chromatograms in a or b is reproduced alongside its mass spectra solely for reference.

c. Peak 1: **$RR(FR)_2$ – R_4F_2:** The composition is derived from the mass, and the retention time matches a product of the $R(FR)_2$ +R, but not the $R(FR)_2$ + r, reactions.
d. Peak 2: **$rR(FR)_2$ – R_4F_2:** The composition is derived from the mass, and the retention time matches a product of the $R(FR)_2$ + r, but not the $R(FR)_2$ + R, reactions.
e. Peak 3: **$R(FR)_2$ – R_3F_2:** The composition is derived from the mass, and therefore it must be the substrate peptide $R(FR)_2$ originally obtained by SPPS.
f. Peak 4: **$fR(FR)_2$ – R_3F_3:** The composition is derived from the mass, and the retention time matches a product of the $R(FR)_2$ + f, but not the $R(FR)_2$ + F reactions.
g. Peak 5: **R_4F_3:** The composition of the peptide is derived from the mass.
h. Peak 6: **R_4F_3**: The composition of the peptide is derived from the mass.
i. Peak 7: **$R(FR)_3$ – R_4F_3:** The composition is derived from the mass and the retention time matches the $R(FR)_3$ peptide obtained by SPPS.
j. Peak 8: **$(FR)_3$ – R_3F_3:** The composition is derived from the mass, and the retention time matches a product of the $R(FR)_2$ + F, but not the $R(FR)_2$ + f reactions.
k. Peak 9: **$ffR(FR)_2$ – R_3F_4:** The composition is derived from the mass, and the retention time matches a product of the $R(FR)_2$ + f, but not $R(FR)_2$ + F reactions.
l. Peak 10: **ff, FF – F_2:** The composition is derived from the mass. The mass spectrum trace in red is a from peak1 to show a contaminant peak that appears in all spectra close to the mass of **F_2**. As expected, the retention times of ff and FF are identical as seen in the $R(FR)_2$ +f and $R(FR)_2$ +F reactions.
m. Peak 11: **$(FR)_4$ – R_4F_4:** The composition is derived from the mass, and the retention time matches a product of the $R(FR)_3$ +F reaction.
cn Peak 12: **$F(FR)_3$ – R_3F_4:** The composition is derived from the mass, and the retention time matches a product of the $R(FR)_2$ +F, but not the $R(FR)_2$ +f reactions.
o. Peak 13: **R_3F_4:** The composition of the peptide is derived from the mass.
p. Peak 14: **R_3F_5:** The composition of the peptide is derived from the mass.
q. Peak 15: **R_3F_4:** The composition of the peptide is derived from the mass.
r. Peak 16: **$FF(FR)_3$ – R_3F_5:** The composition is derived from the mass. This product appears in both the $R(FR)_2/(FE)_4$ + R,F and $R(FR)_2/(FE)_4$ + r,R,f,F reactions, was thus identified as $FF(FR)_3$. However unlikely, the possibility that other peptides with the same composition (diastereomers) co-elute with $FF(FR)_3$ cannot be excluded.
s. Peak 17: **fF, Ff – F_2:** The composition is derived from the mass. The mass spectrum trace in red is a from peak1 to show a contaminant peak that appears in all spectra close to the mass of **F_2**. The retention times of ff and FF are known (peak 10), therefore, the sequence is fF or Ff.

Supplementary Table 1. Yields for single and double additions to **R(FR)$_3$**

Reaction		Yield [%]	
	**(FE)$_4$**	single	double
F	−	2.8	nd
	+	50	nd
V	−	3.2	nd
	+	48	nd
D	−	33	0.7
	+	35	1.8
R	−	2.6	nd
	+	3.5	nd
G	−	13	5.6
	+	30	3.9

Supplementary Table 2. Stereo-selectivity of **R(FR)$_3$/(FE)$_4$** versus **R(FR)$_3$**

amino acid[1]	conc [µM]	**(FE)$_4$**	ratio (L/D)[2]	d.e. (L)[2,3]	fold Δ L/D[4]
DL-V	50	−	0.96	-0.02	5.9
		+	5.7	0.70	
	100	−	0.98	-0.01	6.4
		+	6.2	0.72	
	200	−	0.95	-0.03	7.6
		+	7.2	0.76	
	400	−	1.1	0.03	8.3
		+	8.8	0.80	
DL-L	50	−	0.5	-0.31	4.9
		+	2.6	0.45	
	100	−	0.9	-0.07	3.6
		+	3.1	0.51	
	200	−	1.1	0.04	3.5
		+	3.8	0.58	
	400	−	1.3	0.12	3.4
		+	4.3	0.62	
DL-F	50	−	0.6	-0.28	6.2
		+	3.5	0.55	
	100	−	0.6	-0.28	6.9
		+	3.9	0.59	
	200	−	1.0	0.00	5.9
		+	5.9	0.71	
	400	−	1.5	0.21	5.1
		+	7.9	0.77	
DL-Y	50	−	0.9	-0.07	2.3
		+	2.0	0.34	
	100	−	0.7	-0.18	2.9
		+	2.0	0.34	
	200	−	0.7	-0.16	3.3
		+	2.4	0.41	
	400	−	1.0	-0.02	3.8
		+	3.7	0.58	
DL-W[5]	50	−	0.8	-0.10	3.0
		+	2.4	0.42	
	100	−	1.0	0.00	3.2
		+	3.2	0.52	
	200	−	1.4	0.15	3.6
		+	4.9	0.66	
	400	−	2.3	0.39	3.3
		+	7.5	0.77	

1. Standard single letter abbreviations. DL-A data not included due to heavy peak overlaps (see Supplementary Fig. 2).
2. Only single additions included in the ratio of L- to D- addition products and diastereomeric excess (d.e.).
3. The d.e. values (+ for L-addition) are shaded from red (lowest value) via white to blue (highest value).
4. The ratio of the L/D ratio for the **+(FE)$_4$** reaction to the that of the **-(FE)$_4$** reaction.
5. The tryptophan L- addition product elutes close to di-tryptophan and its peak area was integrated starting from the valley between the peaks to the tail of the peak (see Supplementary Fig. 2). This analysis will underestimate the L- addition product peak area since it is always the larger of the two peaks.

Supplementary Table 3. N-terminal specificity as function of pH[1]

	$(OV)_4$		$(OV)_4$ / $V(DV)_4$		
pH	rel. yield [%] / specificity[2]	Yield[3] [%]	rel. yield [%] / specificity[2]	Yield[3] [%]	Fold Δ specificity[4] amyloid / soluble
8.6	7.2 / 0.3	1.4	53 / 4.6	9.4	14.8
8.0	9.5 / 0.4	1.7	60 / 5.9	10	14.2
7.4	14 / 0.7	2.1	69 / 9.1	9.1	13.8
6.8	19 / 1.0	1.9	78 / 14	8.2	14.5
6.2	21 / 1.1	1.2	82 / 18	8.3	16.7
5.6	25 / 1.3	1.4	84 / 21	8.0	15.2

1. The valine concentration for the reactions was 100 μM and the substrate/template peptides were at 100/120 μM.
2. The relative yield is the amount of N-terminal addition product (**$V(OV)_4$**) as a percent of all single addition products. The specificity is the amount of N-terminal addition product compared to the average amount of each side chain product.
3. The yield is the amount of N-terminal addition product as a percent of the total substrate added to the reaction.
4. The fold change in specificity is the ratio of the N-terminal specificity of the amyloid to that of the soluble peptide.

Supplementary Table 4. N-terminal specificity as function of NaCl concentration[1]

	NaCl [M]	$(OV)_4$ rel. yield / specificity[2]	$(OV)_4$ Yield[3] [%]	$(OV)_4$ / $V(DV)_4$ rel. yield / specificity[2]	$(OV)_4$ / $V(DV)_4$ Yield[3] [%]	Fold Δ specificity[4] amyloid / soluble
no buffer	0	19 / 0.9	4.4	58 / 5.5	15	6.0
	0.1	28 / 1.6	6.1	64 / 7.1	14	4.5
	0.5	34 / 2.1	6.7	65 / 7.5	14	3.6
	1	35 / 2.1	6.9	67 / 8.0	13	3.8
	1.5	36 / 2.3	7.1	68 / 8.5	14	3.8
	2	35 / 2.2	7.1	69 / 9.0	14	4.2
	3	33 / 2	6.7	73 / 11	16	5.2
	4	32 / 1.9	6.4	75 / 12	17	6.5
50mM NaPO4, pH 7.4	0	18 / 0.9	2.7	62 / 6.4	7.1	7.3
	0.1	20 / 1.0	2.9	66 / 7.6	7.1	7.6
	0.5	26 / 1.4	3.0	58 / 5.4	5.2	4.0
	1	31 / 1.8	3.1	67 / 8.1	4.5	4.5
	1.5	33 / 2.0	2.8	67 / 8.3	4.0	4.2
	2	34 / 2.0	2.4	71 / 9.9	3.9	4.9
	3	34 / 2.1	1.9	75 / 12	3.0	5.8
	4	35 / 2.1	1.4	78 / 14	2.9	6.8

1. The valine concentration for the reactions was 100 μM and the substrate/template peptides were at 100/120 μM.
2. The relative yield is the amount of N-terminal addition product (**$V(OV)_4$**) as a percent of all single addition products. The specificity is the amount of N-terminal addition product compared to the average amount of each side chain product.
3. The yield is the amount of N-terminal addition product as a percent of the total substrate added to the reaction.
4. The fold change in specificity is the ratio of the N-terminal specificity of the amyloid to that of the soluble peptide.

Supplementary References

1. Ehler, K.W. & Orgel, L.E. N,N'-carbonyldiimidazole-induced peptide formation in aqueous solution. *Biochim Biophys Acta* **434**, 233-243 (1976).
2. Tycko, R. Physical and structural basis for polymorphism in amyloid fibrils. *Protein Sci* **23**, 1528-1539 (2014).
3. Rapaport, H., Kjaer, K., Jensen, T.R., Leiserowitz, L. & Tirrell, D.A. Two-dimensional order in beta-sheet peptide monolayers. *J. Am. Chem. Soc.* **122**, 12523-12529 (2000).
4. Strohalm, M., Kavan, D., Novak, P., Volny, M. & Havlicek, V. mMass 3: a cross-platform software environment for precise analysis of mass spectrometric data. *Anal Chem* **82**, 4648-4651 (2010).
